# Validation of the GALAD model and establishment of a new model for HCC detection in Chinese patients

**DOI:** 10.3389/fonc.2022.1037742

**Published:** 2022-12-23

**Authors:** Ping Chen, Haolin Song, Wei Xu, Jin Guo, Jianfei Wang, Juhong Zhou, Xiang Kang, Chaolei Jin, Yubo Cai, Zixuan Feng, Hainv Gao, Fengmin Lu, Lanjuan Li

**Affiliations:** ^1^ Department of Infectious Diseases, Shulan (Hangzhou) Hospital Affiliated to Zhejiang Shuren University Shulan International Medical College, Hangzhou, China; ^2^ College of Medicine, Zhejiang University, Hangzhou, China; ^3^ College of Basic Medical Sciences, Zhejiang Chinese Medical University, Hangzhou, China; ^4^ Shulan International Medical College, Zhejiang Shuren University, Hangzhou, China; ^5^ Research and Development Division, Oriomics Biotech Inc, Hangzhou, China; ^6^ Infection and Immunity Institute and Translational Medical Center of Huaihe Hospital, Henan University, Kaifeng, China; ^7^ State Key Laboratory for Diagnosis and Treatment of Infectious Diseases, First Affiliated Hospital, School of Medicine, Zhejiang University, Hangzhou, China; ^8^ National Clinical Research Center for Infectious Diseases, First Affiliated Hospital, School of Medicine, Zhejiang University, Hangzhou, China; ^9^ Collaborative Innovation Centre for Diagnosis and Treatment of Infectious Diseases, First Affiliated Hospital, College of Medicine, Zhejiang University, Hangzhou, China; ^10^ Department of Microbiology & Infectious Disease Center, School of Basic Medical Sciences, Peking University Health Science Center, Beijing, China; ^11^ Hepatology Institute, Peking University People’s Hospital, Beijing, China

**Keywords:** hepatocellular carcinoma, alpha-fetoprotein, DCP, GALAD, GAADPB

## Abstract

**Background:**

GALAD model is a statistical model used to estimate the possibility of hepatocellular carcinoma (HCC) in patients with chronic liver disease. Many studies with other ethnic populations have shown that it has high sensitivity and specificity. However, whether this model can be used for Chinese patients remains to be determined. Our study was conducted to verify the performance of GALAD model in a Chinese cohort and construct a new model that is more appropriately for Chinese populations.

**Methods:**

There are total 512 patients enrolled in the study, which can be divided into training set and validation set. 80 patients with primary liver cancer, 139 patients with chronic liver disease and 87 healthy people were included in the training set. Through the ROC(receiver operating characteristic) curve analysis, the recognition performance of GALAD model for liver cancer was evaluated, and the GAADPB model was established by logistic regression, including gender, age, AFP, DCP, total protein, and total bilirubin. The validation set (75 HCC patients and 130 CLD patients) was used to evaluate the performance of the GAADPB model.

**Result:**

The GALAD and GAADPB achieved excellent performance (area under the receiver operating characteristic curve [AUC], 0.925, 0.945), and were better than GAAP, Doylestown, BALAD-2, aMAP, AFP, AFP-L3%, DCP and combined detection of AFP, AFP-L3 and DCP (AUCs: 0.894, 0.870, 0.648, 0.545, 0.879, 0.782, 0.820 and 0.911) for detecting HCC from CLD in the training set. As for early stage of HCC (BCLC 0/A), GAADPB had the best sensitivity compared to GALAD, ADP and DCP (56.3%, 53.1%, 40.6%, 50.0%). GAADPB had better performance than GALAD in the test set, AUC (0.896 vs 0.888).

**Conclusions:**

The new GAADPB model was powerful and stable, with better performance than the GALAD and other models, and it also was promising in the area of HCC prognosis prediction. Further study on the real-world HCC patients in China are needed.

## Introduction

With approximately 906,000 new cases and 830,000 deaths globally in 2020, hepatocellular carcinoma (HCC) was known as the sixth most diagnosed cancer and the third leading cause of cancer-related mortality (1).The etiology of HCC is geographically diversified. Worldwide, most cases are related to HBV infection, while HCV infection is the most common case of HCC in some Western countries as well as Japan (2). In China, HBV infection is the leading cause of HCC (3). Surgical resection, ablation, or liver transplantation can be used to cure early-stage HCC. However, those patients who are diagnosed at an advanced stage have limited access to treatments and a poor prognosis (3). A lot of patients in China are already at an advanced stage at the time of diagnosis. Given that situation, early surveillance of HCC among high-risk population is of great importance.

For better surveillance of high-risk groups, abdominal ultrasound is recommended every six months, with or without alpha-fetoprotein (AFP) serum test. However, the test results are operator-dependent. The sensitivity could vary from 47% to 84% (4). Further, obesity might affect the performance of ultrasound (5). As a surveillance test, ultrasound still has some limitation, so AFP is often used in combination with ultrasound. While detection rate can be increased by combining these two methods, there is also an increased suspicion of false-positive and cost (6). Besides, patients with chronic viral hepatitis can also have an elevated alpha-fetoprotein (5). To monitor HCC more accurately at an early stage,other serum-based biomarkers, such as Lens culinaris agglutinin-reactive fraction of AFP (AFP-L3), and Des-gamma-carboxy prothrombin (DCP), have been put into use (7). However, none of the single serum marker for early surveillance of HCC has been proved to meet the clinical demands (8, 9).

The GALAD score, as well as Doylestown, BALAD and aMAP, which is derived from gender, age, AFP, AFP-L3, DCP, bilirubin, platelets and albumen, has showed high sensitivity for HCC detection in reported studies (9, 10). However, the studied samples of these reports had characteristics different from those of Chinese HCC patients, where hepatitis B virus (HBV) infection is the most common etiology of HCC in China whereas HCV infection was the main reason in other ethnic populations.

This study aimed to assess the performance of the GALAD model for HCC diagnosis and explore a better model for Chinese patients.

## Materials and methods

### Study populations

This study enrolled 307 participants in training set, consisting of 80 HCC patients, 140 non-cancer controls with CLD [100 patients with liver cirrhosis (LC) and 40 patients with chronic hepatitis B (CHB)] and 87 healthy controls (HC). In testing set, a total of 205 participants was enrolled, including 75 HCC patients and 130 CLD controls (106 LC/24 CHB). All participants were recruited between 2019 and 2021 from Shulan hospital in Hangzhou. For all patients, the diagnosis was established by the histologic examination of tumor tissue or characteristic medical imaging including computed tomography (CT), magnetic resonance imaging (MRI), according to clinical practice guidelines. We collected information on 29 clinical characteristics and other variables for all participants, consisting of age, gender, etiology of liver disease, laboratory results, and baseline tumor characteristics at the time of diagnosis (**Tables 1**, **S1**). Written informed consent was obtained from every participant and the study protocol was approved by the Ethics Committee of Shulan hospital.

**Table 1:** Characteristics of the study participants used to develop the GAADPB model.

Variables	HCC (n = 80)	CLD			HC (n = 87)	*P* value (HCC vs CLD)
		Total(n = 140)	LC(n = 100)	CHB(n = 40)		
**Demographics**						
Age, y, median (interquartile range)	54.5 (47-63.3)	52 (43~58.3)	54 (47~61)	44.5 (39~52.3)	39 (32.5~48)	0.021^*^
Male sex, n (%)	74 (92.5%)	106 (75.7%)	75 (75%)	31 (77.5%)	46 (52.9%)	0.002^**^
**Etiology, n (%)**						
HBV	68 (85%)	107 (76.4%)	67 (67%)	40 (100%)	0 (0%)	N/A
Fatty liver	0 (0%)	18 (12.9%)	6 (6%)	0 (0%)	0 (0%)	N/A
Alcohol	5 (6.3%)	19 (13.6%)	18 (18%)	0 (0%)	0 (0%)	N/A
Others	7 (8.75%)	9 (6.4%)	9 (9%)	0 (0%)	0 (0%)	N/A
**HCC biomarkers, median (interquartile range)**						
AFP, ng/ml	42.6 (8.8-820.4)	3.1 (2~5.5)	3.4 (2.1~6.3)	2.4 (1.9~3.8)	2 (1.6~2.6)	<0.001^***^
DCP, ng/ml	143.4 (27.7-5247)	17.5 (11.4~26.9)	20 (12.8~34.3)	14 (9.9~21.4)	7.8 (5.8~11.7)	<0.001^***^
Log_10_AFP	1.6 (0.9-2.9)	0.5 (0.3~0.7)	0.5 (0.3~0.8)	0.4 (0.3-0.6)	0.3 (0.2-0.4)	<0.001^***^
Log_10_DCP	2.2 (1.4-3.7)	1.2 (1.1~1.4)	1.3 (1.1~1.5)	1.1 (1-1.3)	0.9 (0.8-1.1)	<0.001^***^
**Liver function tests, median (interquartile range)**						
TB, μmol/L	17.5 (11.8~25)	18 (12~30)	21 (13~35.3)	12 (10.5~18.5)	13 (11~17)	0.246
TP, g/L	66.7 (63.2~70.3)	69.1 (63.2~73.6)	69 (60.8~73.7)	70.1 (65.8~73)	72.1 (69.9~74)	0.06
**Tumor Characteristics, n (%)**		N/A	N/A	N/A	N/A	N/A
Tumor stage (BCLC)						
0	11 (13.8%)					
A	22 (27.5%)					
B	12 (15%)					
C	31 (38.8)					
D	4 (5%)					
Tumor size						
<3cm	24 (30.0%)					
≥3 and ≤ 5cm	18 (22.5%)					
>5cm	27 (33.8%)					
NA	11 (13.8%)					
Number of tumors						
single	35 (43.8%)					
multiple	43 (53.8%)					
NA	2 (2.5%)					
PPVT						
absent	54 (67.5%)					
present	26 (32.5%)					
Metastasis						
absent	70 (87.5%)					
present	10 (12.5%)					

All continuous variables were presented as median (interquartile range); Categorical variables were presented as frequencies (percentage). HCC, hepatocellular carcinoma; CLD, chronic liver disease; LC, liver cirrhosis; CHB, chronic hepatitis B; HC, healthy controls; HBV, hepatitis B virus; AFP, alpha-fetoprotein; AFP-L3, lens culinaris agglutinin-reactive alpha-fetoprotein; DCP, des-gamma-carboxy prothrombin; TP. total protein; TB, total bilirubin; PPVT, portal vein tumor thrombus; NA, not available; N/A, not applicable. *P <0.05, **P<0.01, ***P <0.001.

### Inclusion criteria

HCC patients were diagnosed by referring “Guidelines for the diagnosis and treatment of primary liver cancer”: 1) Histopathological diagnosis; or 2) Medical imaging diagnosis, includes of computed tomography (CT), magnetic resonance imaging (MRI), Contrast enhanced-ultrasonography(CEUS) or Gd-EOB-DTPA-MRI(EOB-MRI): A) if liver had a nodule equal or less than 2 cm, and more than 2 medicals imaging results revealing typical imaging lesions of HCC; B) if liver had a nodule greater than 2 cm, and more than 1 medical imaging results revealing typical imaging lesions of HCC; or C) if liver had not nodule, but AFP values was positive and more than 1 medical imaging results revealing typical imaging lesions of HCC. CHB patients were diagnosed by referring “The guideline for the prevention and treatment of CHB infection from the Chinese Society of Hepatology”: HBV infection more than 6 months, alanine aminotransferase (ALT) is persistently or repeatedly elevated, or hepatitis lesions are identified by liver biopsy. LC patients were diagnosed by referring “Chinese guidelines on the management of liver cirrhosis”: 1) Histopathological diagnosis; or 2) Medical imaging diagnosis, Ultrasound(US), CT or MRI imaging results revealing splenomegaly without liver space-occupying lesions. Healthy participants were a group of people with normal physical examination in Shulan Hospital: 1) No family history of cancer, no history of liver disease diagnosis and treatment; (2) no HBV or HCV infection; 3) liver function, renal function, and routine blood tests were normal; 4) Ultrasound results were normal in the liver or gallbladder system; and (5) liver fiber scan results were normal.

### Exclusion criteria

(1) Subjects with HCC with other tumors; (2) Subjects who cannot be sampled, have insufficient sample size or have unqualified samples; (3) Subjects with liver metastases or HCC treatment (such as: surgery, ablation, radiotherapy or chemotherapy).

### AFP, AFP-L3%, and DCP assay

About 10 mL peripheral blood was collected from each participant. For HCC and CLD patients, blood sample was drawn prior to the treatment. Serum AFP, AFP-L3%, and DCP were assayed by Hotgen Biotech Co., Ltd (Beijing, China) by using chemiluminescence microparticle immunoassay. The quantitative limit of AFP, AFP-L3, AFP-L3%, and DCP was 0.6-20000ng/mL, 0.6-20000ng/mL, 5–50% and 0.6-20000ng/mL, respectively. If these biomarker values exceeded extreme ones, we used extreme values to represent. On the other hand, if both AFP and AFP-L3 exceeded extreme values, we then used 10% (AFP-L3/AFP) to represent AFP-L3% positive.

### Performance evaluation of five models for the discrimination of HCC from CLD

We compared five previous developed models for assessing their discrimination ability of HCC from CLD in our sample. GALAD score was calculated based on five variables (age, gender, AFP, AFP-L3 and DCP) (11). GAAP score was calculated on the basis of four variables (age, gender, AFP and DCP) (12). Doylestown score was calculated based on five variables (age, gender, AFP, ALP and ALT) (13). BALAD-2 score was calculated based on five variables (bilirubin, total albumin, AFP, AFP-L3 and DCP) (14). aMAP score was calculated based on five variables (age, gender, total bilirubin, albumin and PLT) (15). **Table S2** shows the detailed equations of these models. Cutoff values of these models were based on published thresholds (GALAD: -0.63, GAAP: -0.65, Doylestown: 0.5, BALAD-2: 0.66, and aMAP: 60). Performance of these models was performed by calculating the area under each receiver operating characteristic (ROC) curve.

### Model development

Logistic regression for multivariate analysis was used to assess the association of the 20 variables, including demographics, HCC biomarkers, liver function, blood routine test, and blood coagulation parameters; see **Tables 1**, **S1**) with HCC based on the use of a forward-backward stepwise approach. Participants with missing data were excluded from the statistical analysis. Logarithmic transformation of AFP, AFP-L3 and DCP values was applied for logistic regression analysis due to extreme skewness (11). Age has been considered as a good variable for HCC risk assessment (11, 14, 15). Therefore, we built a new HCC risk assessment model, called GAADPB based on variables (gender, AFP, DCP, TP and TB) selected from multivariate analyses and age. The formula was shown as follows:

GAADPB score = 0.176 + 0.162*gender +0.002*age +0.178*log_10_AFP+0.164*log_10_DCP-0.007*TP-0.002*TB

The probability of having HCC was calculated using the following formula: P(HCC) = exp[score]/(1 + exp[score]).

### Statistical analysis methods

SPSS (version 22.0) software (IBM/SPSS Inc., Chicago, IL) or R language (version 3.4.4) were used to perform the statistical analysis and draw figures. Continuous variables were presented as medians (interquartile range) and categorical variables were presented as frequencies (percentage). Characteristics differences were tested using Wilcoxon test for continuous variables and Chi-square tests for categorical variables. SPSS software was used to analyze ROC curve. The results of sensitivity, specificity, and AUC were used to reveal models/biomarkers performance. Comparisons of ROC curves were performed using the “roc.test” function in pROC package in R language (parameter paired = “TRUE” and method = “delong”). All statistical tests were two-sided. *P* value ≤0.05 was considered to be statistically significant.

## Results

### Demographic data and clinical characteristics of enrolled participants in training set

A total of 307 participants were enrolled in the training set, including 80 HCC patients, 140 CLD (100 HC/40 CHB) and 87 HC participants. The demographic data, clinical characteristics and laboratory results of the study participants were described in **Tables 1**, **S1**. HCC patients were significantly older than those with chronic liver disease (median 54.5 years vs 52 years; *P*=0.021), and the proportion of males in HCC patients were significantly higher compared to that in CLD population (92.5% vs 75.7%, *P*=0.002). In addition,41.3% HCC patients were at a very early/early stage (BCLC 0/A).

### Screening candidate biomarkers for model construction

To find potential biomarkers that can be used to distinguish HCC patients from non-HCC controls among Chinese population, two approaches were employed. Firstly, we applied logistic regression analysis to screen independent factors associated with HCC. After excluding one CHB patient due to incomplete data, we finally included 80 HCC, 100 LC and 39 CHB samples for logistic regression analysis, which contained 20 variables from demographic characteristics, routine blood test, liver function test, and blood clotting test (**Tables 1**, **S1**). The results demonstrated that gender, AFP, DCP, TP and TB were independent factors for HCC (**Table 2**).

**Table 2:** Parameter estimation of variables by logistic regression in GAADPB model.

Variable	Estimate	Standard Error	t value	*P* value
Constant	0.176	0.282	0.626	0.532
Age	0.002	0.002	0.861	0.391
Gender	0.162	0.063	2.583	0.011^*^
Log_10_AFP	0.178	0.027	6.677	<0.001^***^
Log_10_DCP	0.164	0.031	5.316	<0.001^***^
TB	-0.002	0.001	-2.857	0.005^**^
TP	-0.007	0.003	-2.131	0.034^*^

AFP, alpha-fetoprotein; DCP, des-gamma-carboxy prothrombin; TP, total protein; TB, total bilirubin. *P <0.05, **P<0.01, ***P <0.001.

**Figure 1:**
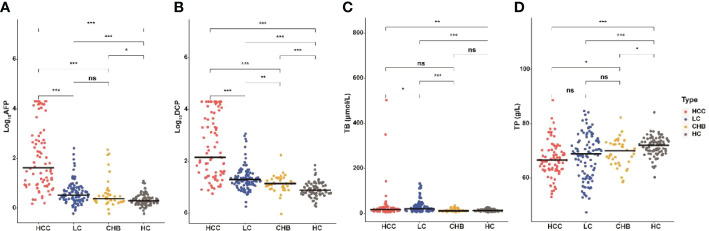
Serum AFP, DCP, TB and TP in HCC and non-HCC groups. Comparison of AFP **(A)**, DCP **(B)**, TB **(C)**, and TP **(D)** among HCC, LC, CHB, and HC groups. The horizontal bar represents median values. Characteristics differences were tested using Wilcoxon test. ns *P*>0.05, *P <0.05, **P <0.01, ***P <0.001. HCC, hepatocellular carcinoma; LC, liver cirrhosis; CHB, chronic hepatitis B; HC, healthy controls; TP, total protein; TB, total bilirubin.

We also compared the levels of 18 blood indicators between HCC patients and patients at high-risk for HCC (CLD group; **Tables 1**, **S1**) to screen significantly difference indicators. According to our samples, the mean values of AFP, DCP, AFP-L3, WBC, PLT, ALT, AST, ALP, GGT and ALB had significant difference between HCC and CLD groups (*P*<0.05). We further compared these variables between different disease subgroups (**Figures 1**, **S1**), and found that the levels of AFP, DCP, AFP-L3, AST and GGT in HCC group were significantly higher than that in LC and CHB subgroups. However, TP, RBC, HGB, ALP, DBIL, ALB, A/G, PT and INR had a similar level between HCC and LC group, and TB, WBC and ALT could not discriminate HCC from CHB group. In addition, the level of PLT in HCC group were significantly higher than that in LC group, but significantly lower than that in CHB and HC groups. These results suggested that the AFP, DCP, AFP-L3, AST and GGT biomarkers might be able to potentially discriminate HCC patients from non-HCC patients.

### Development of a new HCC risk assessment model

In order to build a more suitable HCC diagnostic model, we referred to the ideas developed by GALAD (16). HCC and high-risk groups for HCC were used in the construction of a diagnostic model for HCC. We included above identified independent factors, gender combined with blood indicators showing significant difference between HCC and CLD groups, and all candidate biomarkers obtained from two approaches in a multivariate model to construct models 1, 2 and 3, respectively. The performance of each model is showed in **Table 3**. The AUC of models 1, 2 and 3 was 0.941, 0.927 and 0.94, respectively. These results suggested that, although the values of AFP-L3, AST and GGT alone were significantly different between HCC and non-HCC groups, these variables were not effective in improving the performance of HCC diagnostic models. Furthermore, we found that the age variable was filtered out by logistic regression analysis. However, numerous studies (15–17)had shown that age variable can be enrolled into models related to HCC, and “Guidelines for the diagnosis and treatment of primary liver cancer” also suggested that men more than 40 years old is also part of the high-risk group for HCC. Therefore, we enrolled the age variable into model 1 and developed a new model for HCC diagnosis, GAADPB. The performance of GAADPB (AUC=0.941, **Table 3**) was similar to model 1 and slightly higher than GALAD (P= 0.0545).

**Table 3:** Models performance for discriminating HCC (n = 80) and CLD (n = 139) (Specificity=90%).

Model	AUC	95% CI	Sensitivity %
GALAD	0.925	0.89-0.96	81.3
Model 1	0.941	0.907-0.975	86.3
Model 2	0.927	0.888-0.967	86.3
Model 3	0.94	0.905-0.975	87.5
GAADPB	0.941	0.908-0.974	87.5

Model 1 consists of Gender, Log_10_AFP, Log_10_DCP, TP and TB; Model 2 consists of Gender, Log_10_AFP, Log_10_DCP, Log_10_AFP-L3, AST and GGT; Model3 consists of Gender, Log_10_AFP, Log_10_DCP, Log_10_AFP-L3, AST, GGT, TP and TB. GAADPB consists of Gender, age, Log_10_AFP, Log_10_DCP, TP and TB; AUC, area under receiver operating characteristic curve; HCC, hepatocellular carcinoma; CLD, chronic liver disease.

### Subgroup analysis of model performance

**Figure 2:**
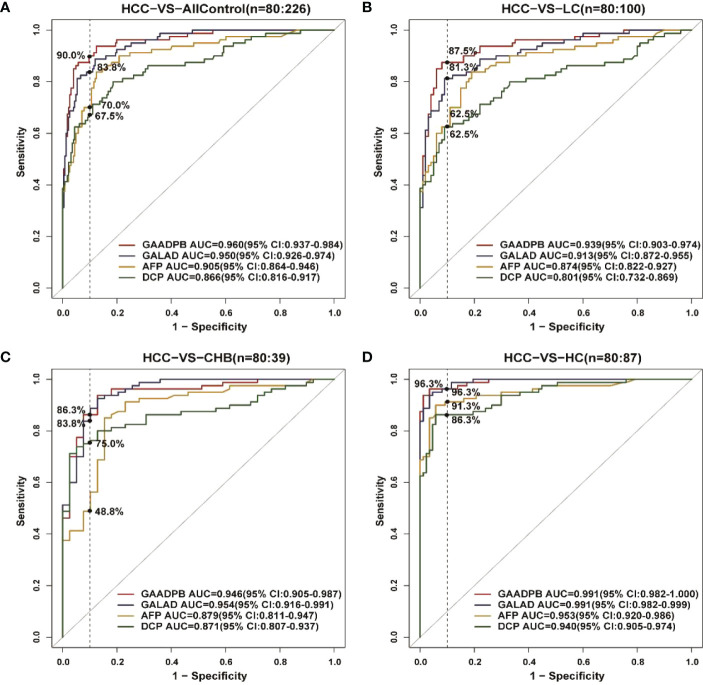
ROC curves comparing performance among GAADPB, GALAD and individual protein biomarkers for discriminating HCC from different disease subgroups and healthy controls. **(A)**, HCC from all control **(B)**, HCC from LC **(C)**, HCC form CHB **(D)**, HCC form HC. The vertical line represents specificity at 90%. HCC, hepatocellular carcinoma; LC, liver cirrhosis; CHB, chronic hepatitis B; HC, healthy controls.

We analyzed the performance of GAADPB for differentiating HCC from different disease subgroups (training set) and health controls in our sample, and the results are shown in **Figure 2** and **Table 4**. The AUC of GAADPB for differentiating HCC from LC was 0.939, which was significantly higher than that of GALAD (AUC_LC_=0.913; *P*=0.01), AFP (AUC_LC_=0.874; *P*=0.001) and DCP (AUC_LC_=0.801; *P <*0.001). In addition, the AUC of GAADPB for differentiating HCC from CHB and HC subgroups was 0.946 and 0.991, respectively, which was similar to the performance of GALAD (AUC_CHB_=0.954, *P*=0.453; AUC_HC_=0.991, *P*= 0.857) and significantly higher than that of AFP (AUC_CHB_= 0.879, *P*=0.003; AUC_HC_=0.953, *P*=0.005) and DCP (AUC_CHB_=0.871, *P*=0.009; AUC_HC_=0.94, *P*=0.001). Collectively, the performance of GAADPB for different subgroups was significantly better than that of a single protein biomarker, and it was more suitable than GALAD for distinguishing HCC from LC patients and similar to GALAD for distinguishing HCC from CHB and HC.

**Table 4:** Comparison between GAADPB, GALAD and the individual biomarkers within disease subgroups (Specificity=90%).

	All Control			LC			CHB			HC		
		AUC(95%CI)	Sen%	*P* value	AUC(95%CI)	Sen%	*P* value	AUC(95%CI)	Sen%	*P* value	AUC(95%CI)	Sen%	*P* value
GAADPB	0.96 (0.937-0.984)	90.0	–	0.939 (0.903-0.974)	87.5	–	0.946 (0.905-0.987)	86.3	–	0.991 (0.982-1)	96.3	–
GALAD	0.95 (0.926-0.974)	83.8	0.088	0.913 (0.872-0.955)	81.3	0.01^*^	0.954 (0.916-0.992)	83.8	0.453	0.991 (0.982-1)	96.3	0.857
AFP	0.905 (0.864-0.946)	70.0	<0.001^***^	0.874 (0.822-0.927)	62.5	0.001^**^	0.879 (0.812-0.947)	48.8	0.003^**^	0.953 (0.92-0.986)	91.3	0.005^**^
DCP	0.866 (0.816-0.917)	67.5	<0.001^***^	0.801 (0.732-0.869)	62.5	<0.001^***^	0.871 (0.807-0.934)	75.0	0.009^**^	0.94 (0.905-0.974)	86.3	0.001^**^

All Control include of LC, CHB and HC; P value derived from GAADPB VS others. AUC, area under receiver operating characteristic curve; hepatocellular carcinoma; LC, liver cirrhosis; CHB, chronic hepatitis B; HC, healthy controls; AFP, alpha-fetoprotein; DCP, des-gamma-carboxy prothrombin; Sen, Sensitivity. *P <0.05, **P<0.01, ***P <0.001.

**Figure 3:**
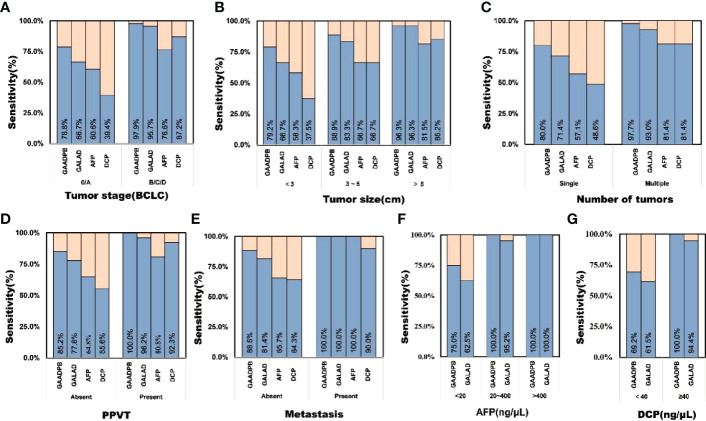
Sensitivity comparison of GAADPB and GALAD at 90% specificity in different cancer subgroups. **(A)**, Tumor stage (BCLC) **(B)**, Tumor size **(C)**, Number of tumors **(D)**, PPVT **(E)**, Metastasis **(F)**, AFP **(G)**, DCP. HCC, hepatocellular carcinoma; LC, liver cirrhosis; CHB, chronic hepatitis B; HC, healthy controls, PPVT, portal vein tumor thrombus.

In addition, we analyzed the performance of GAADPB in different cancer subgroups in training set. GAADPB performed significantly better for distinguishing HCC than individual protein biomarkers in many HCC subgroups and was almost identical to GALAD (**Figure 3** and **Table S3**). As a diagnostic model, it is necessary to maximize the detection rate of the model under the premise of avoiding excessive medical treatment. Therefore, we compared the sensitivity of GAADPB and GALAD at a specificity of 90%. The results showed that GAADPB appeared more sensitive than GALAD for detecting different HCC subgroups with very early/early stage (BCLC 0/A), small size (diameter < 3 cm), single lesion, absent PPVT, absent metastases, AFP-negative (20 ng/ml) and DCP-negative (<40 ng/ml), especially in (very) early-stage (BCLC 0/A), small size (diameter< 3 cm) and AFP-negative HCC, which improved its sensitivity by 12.1%, 12.5%, and 12.5% than GALAD, respectively. These results suggested that GAADPB performed even better in detecting HCC compared to GALAD in a more subtle situation.

### Performances of GAADPB in testing set

**Figure 4:**
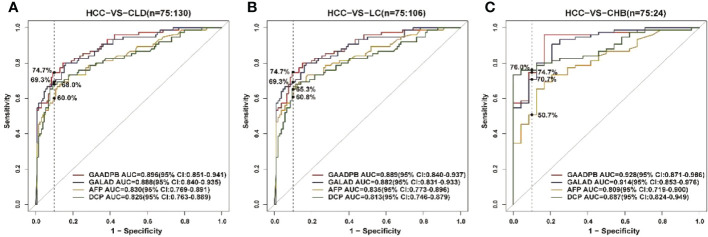
ROC curve analysis of GAADPB in different disease subgroups of the test set. **(A)**, HCC from CLD **(B)**, HCC from LC **(C)**, HCC form CHB. The vertical line represented specificity was at 90%. HCC, hepatocellular carcinoma; LC, liver cirrhosis; CHB, chronic hepatitis B; HC, healthy controls.

**Figure 5:**
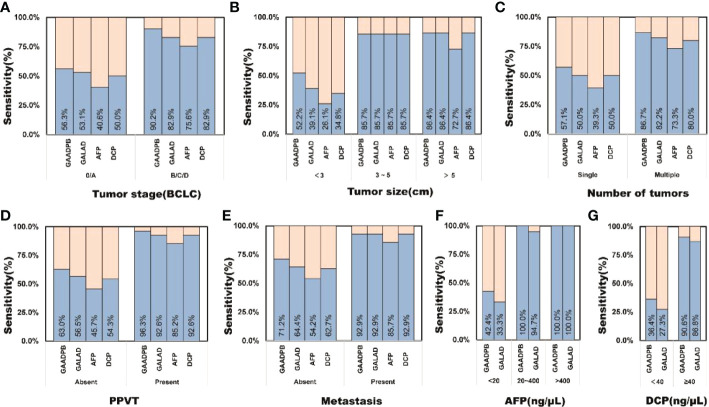
The sensitivity of GAADPB in different cancer subgroups of test set. **(A)**, Tumor stage (BCLC) **(B)**, Tumor size **(C)**, Number of tumors **(D)**, PPVT **(E)**, Metastasis **(F)**, AFP **(G)**, DCP. HCC, hepatocellular carcinoma; LC, liver cirrhosis; CHB, chronic hepatitis B; HC, healthy controls, PPVT, portal vein tumor thrombus.

To evaluate the stability of GAADPB in distinguishing HCC from high-risk population. We included an independent testing set for model validation. The information for demographic data and clinical characteristics of the participants in the testing set are presented in **Table 5**. In the testing set, the AUC of GAADPB was 0.896 for distinguishing HCC from CLD patients, and the sensitivity was 76.7% at a specificity of 90% (**Figure 4**). The preformation for different disease subgroups is showed in **Figure 4**. The AUC of GAADPB was 0.889 and 0.928 for distinguishing HCC from LC and CHB, respectively. When the specificity was 90%, the sensitivity was 74.7% in both LC and CHB subgroups. Furthermore, we assessed GAADPB performance for distinguishing HCC from different cancer subgroups, the results revealed that at a 90% specificity, GAADPB still had the highest sensitivity compared to the individual biomarkers for detecting HCC subgroups with very early/early stage (BCLC 0/A), small size (diameter < 3 cm), single lesion, absent PPVT, absent metastases, AFP-negative (20 ng/ml) and DCP-negative (<40 ng/ml)] (**Figure 5** and **Table S4**). These results indicated that GAADPB model was a stable and robust diagnostic tool for differentiating HCC from high-risk population.

**Table 5:** Characteristics of patients used to test the GAADPB model.

Variables	HCC (n = 75)	CLD			*P* value
		Total (n = 130)	LC (n = 106)	CHB (n = 24)	(HCC vs CLD)
**Demographics**					
Age, y, median (interquartile range)	55 (45.5~64)	53 (45~58)	53 (45.3~59)	52 (43.5~57)	0.326
Male sex, n (%)	66 (88%)	94 (72.3%)	78 (73.6%)	16 (66.7%)	0.009^**^
**Etiology, n (%)**					
HBV	57 (76%)	97 (74.6%)	73 (68.9%)	24 (100%)	N/A
Fatty liver	2 (2.7%)	1 (0.7%)	1 (0.9%)	0 (0%)	N/A
Alcohol	4 (5.3%)	18 (13.8%)	18 (17%)	0 (0%)	N/A
Others	12 (16%)	14 (10.8%)	14 (13.2%)	0 (0%)	N/A
**HCC biomarkers, median (interquartile range)**					
AFP, ng/ml	39.2 (5.9~1799.3)	2.9 (2.1~6)	2.9 (2.1~6)	2.9 (2.1~6.1)	<0.001^***^
DCP, ng/ml	305.2 (28.5~10913.5)	17.8 (12.6~25.5)	18 (13.8~29.4)	16.2 (10.8~19.8)	<0.001^***^
Log_10_AFP	1.6 (0.8~3.2)	0.5 (0.3~0.8)	0.5 (0.3~0.8)	0.5 (0.3~0.8)	<0.001^***^
Log_10_DCP	2.5 (1.5~4)	1.2 (1.1~1.4)	1.3 (1.1~1.5)	1.2 (1~1.3)	<0.001^***^
**Liver function tests, median (interquartile range)**					
TB, μmol/L	18 (12~28)	21 (13.3~39.8)	22 (14.3~43.8)	14.5 (10.8~20.3)	0.076
TP, g/L	67 (62.6~72.2)	67.1 (62.2~72.1)	66.8 (61.8~70.5)	69.7 (66.2~75.5)	0.922
**Tumor Characteristics, n (%)**		N/A	N/A	N/A	N/A
Tumor stage (BCLC)					
0	8 (10.7%)				
A	24 (32%)				
B	15 (20%)				
C	25 (33.3%)				
D	1 (1.3%)				
NA	2 (2.7%)				
Tumor size					
<3cm	23 (30.7%)				
≥3 and ≤ 5cm	7 (9.3%)				
>5cm	22 (29.3%)				
NA	23 (30.7%)				
Number of tumors					
single	28 (37.3%)				
multiple	45 (60%)				
NA	2 (2.7%)				
PPVT					
absent	46 (61.3%)				
present	27 (36%)				
NA	2 (2.7%)				
Metastasis					
absent	59 (78.7%)				
present	14 (18.7%)				
NA	2 (2.7%)				

All continuous variables were presented as median (interquartile range); Categorical variables were presented as frequencies (percentage). HCC, hepatocellular carcinoma; CLD, chronic liver disease; LC, liver cirrhosis; CHB, chronic hepatitis B; HC, healthy controls; HBV, hepatitis B virus; AFP, alpha-fetoprotein; AFP-L3, lens culinaris agglutinin-reactive alpha-fetoprotein; DCP, des-gamma-carboxy prothrombin; TP. total protein; TB, total bilirubin; PPVT, portal vein tumor thrombus; NA, not available; N/A, not applicable. **P < 0.01, ***P < 0.001.

## Discussion

About 350 million people are infected with HBV globally, and the lifetime risk of developing HCC among HBV carriers ranges from 10% to 25% (18). Most HCC cases in China are related to HBV infection, which is the same in our study population (19). Currently, the methods based on ultrasound and AFP are not sensitive enough to detect early HCC, so a more effective, objective and accurate Chinese population monitoring method is in need.

Several HCC risk predictions scoring systems had been developed for estimating the risk of HCC development from CHB, diagnose the ability of HCC from CLD, stage HCC and so on (11–13). Since they were all HCC-related models, we wanted to determine whether all these models could be used for the diagnosis of HCC. Therefore, we evaluated and compared the performance of these five models as well as three protein biomarkers for the diagnosis of HCC in our training sample. Our results showed that GALAD had the highest accuracy for HCC detection, with an area under the receiver operating characteristic curve (AUC) of 0.925 (**Table 6**). In addition, combined detection of AFP, AFP-L3 and DCP (AUC=0.91) and GAAP (AUC=0.89) could also better distinguish the HCC patients from the non-HCC population. However, BALAD-2 and aMAP could not predict HCC with a relative high performance, even with significantly lower AUC than individual protein markers (AUC_AFP_=0.876, P_BALAD-2_ VS _AFP_ <0.001, P_aMAP_ VS _AFP_ <0.001), which may be related to the scenario of model development (15, 17).

**Table 6:** ROC curve analysis of HCC prediction models and serum biomarkers for discriminating HCC (n=80) and CLD groups (n=140).

Model/Biomarker	AUC	95%CI	Cut-Off Value	Sensitivity (%)	Specificity (%)	*P* value (GALAD vs Others)
GALAD	0.925	0.89~0.96	-0.63	95	57.1	/
GAAP	0.894	0.852~0.937	-0.65	77.5	84.3	0.006^**^
Doylestown^a^	0.87	0.823~0.917	0.5	67.5	86.3	<0.001^***^
BALAD_2^a^	0.648	0.575~0.72	0.66	30	77.7	<0.001^***^
aMAP^a^	0.545	0.467~0.623	60	57.5	51.8	<0.001^***^
AFP	0.876	0.827~0.926	20	60	92.1	0.006^**^
AFP	0.876	0.827~0.926	400	33.8	100	0.006^**^
DCP	0.821	0.757~0.885	40	67.5	85	<0.001^***^
AFP-L3%	0.783	0.724~0.841	10	56.3	96.4	<0.001^***^
AFP+ DCP+ AFP-L3%^b^	0.911	0.866~0.956	Same as above	87.5	79.3	0.257

^a^one CHB patient was excluded from the analysis; ^b^A positive result was recorded if any of the markers exceeded their specified cut-off point. AUC, area under receiver operating characteristic curve; HCC, hepatocellular carcinoma; CLD, chronic liver disease; AFP, alpha-fetoprotein; AFP-L3, lens culinaris agglutinin-reactive alpha-fetoprotein; DCP, des-gamma-carboxy prothrombin. **P < 0.01, ***P < 0.001.

In order to build a more suitable HCC diagnostic model, we referred to the ideas developed by GALAD and constructed a new model for HCC diagnosis, the GAADPB model (11). Differing from GALAD, in our multivariate logistic regression analysis, total protein (TP) and total bilirubin (TB) are independent factors associated with the developing of HCC, they reflect the synthetic function and the underlying liver function and they are also included in the BALAD-2 and aMAP (11, 12). We excluded AFP-L3, because the contribution of AFP-L3 was small in our study, and the GAAP study had the same results (12). The age variable was filtered out by logistic regression analysis, but according to previous reported studies, the age variable is associated with the incidence of HCC and it is included in many models (11, 13, 15). Therefore, we included the age variable in our model. The performance of GAADPB (AUC=0.941) was better than GALAD (AUC=0.925), GAAP (AUC=0.894) and other models according to our training set. And in the validation set, the performance of GAADPB (AUC=0.896) was also better than the GALAD model (AUC=0.888) and single serum marker. We also assessed GAADPB performance for distinguishing HCC from different cancer subgroups, the results revealed that at 90% specificity, GAADPB still had the highest sensitivity compared to the individual biomarkers and GALAD to detect HCC subgroups. These results suggested that GAADPB model is a stable and robust diagnostic tool to distinguish HCC from high-risk groups.

Despite the poor performance of some models in our training set, these models have shown excellent results in predicting prognosis of HCC. A study based on 3700 HCC patients from Japan and the UK showed that patients with different BALAD scores might have a different prognosis. Patients with lower BALAD scores tended to show worse prognostic features (20). Similarly, a multicenter study by Berhane et al. demonstrated that the BALAD-2 model could, to some extent, predict the survival months in HCC patients (8). Besides, the aMAP score had been shown to be strongly related to HCC development in patients with chronic hepatitis, and late recurrence after radiofrequency ablation of HBV-related HCC patients (21). In our current study, the GAADPB model had a higher AUC in the diagnosis of liver cancer compared to BALAD-2 and aMAP, and GAADPB introduced indicators of liver injury such as TB compared to GALAD, which implied the performance of GAADPB in the areas of liver cancer progression, recurrence and prognosis could be promising in the future.

Early diagnosis of HCC for detecting HCC has been a research hotspot recently. Lots of new technologies such as multitarget HCC blood test (mt-HBT) and liquid biopsies were used to improve early cancer detection, but at the same time they come at a higher cost (22). Our results suggested that GAADPB model had higher sensitivity compared to the individual biomarkers (**Figure 3**), when we were detecting HCC subgroups with very early/early stage (BCLC 0/A), small size (diameter < 3 cm), single lesion, absent PPVT, absent metastases, AFP-negative (20 ng/ml) and DCP-negative (<40 ng/ml). And the performance was confirmed to be stable in the testing set. Therefore, when it comes to early stage HCC that are difficult to diagnose by imaging and traditional biomarkers, GAADPB might be an economic method of auxiliary diagnosis.

This study had some limitations. First of all, in our training set, age did not show a significant correlation with HCC occurrence, but previous reports had demonstrated a correlation between age and HCC occurrence, which may be related to the relatively insufficient sample size compared to the number of independent variables in the multivariate analyses (11, 13, 15). We plan to increase the sample size and make further validation and correction of the GAADPB model to improve the reliability of our findings in future. Besides, this is a retrospective design-based study with data from a single medical center. And the diagnostic performance of DCP was higher than that of Doylestown in the validation set (AUC of DCP: 0.884, 95% CI: 0.841-0.927; Doylestown: 0.845, 95% CI: 0.794-0.896; P <.05). Our further study will focus on validating the GAADPB model based on the real-world HBV-caused HCC in Southeast China.

## Conclusions

In sum, the performance of GALAD in discriminating HCC in CLD people of China was excellent. Our GAADPB model, which also enrolled TB and TP, had better performance than GALAD, especially in detecting early stage HCC. GAADPB was also promising in predicting the prognosis of HCC patients. Further study is needed to proving the function of our GAADPB model in Chinese patients.

## Data availability statement

The original contributions presented in the study are included in the article/**Supplementary Material**. Further inquiries can be directed to the corresponding author.

## Ethics statement

The studies involving human participants were reviewed and approved by Shulan Hangzhou Hospital. Written informed consent for participation was not required for this study in accordance with the national legislation and the institutional requirements.

## Author contributions

All authors listed have made a substantial, direct, and intellectual contribution to the work, and approved it for publication.
