# Corrigendum: Validation of the GALAD model and establishment of a new model for HCC detection in Chinese patients

**DOI:** 10.3389/fonc.2023.1170066

**Published:** 2023-04-19

**Authors:** Ping Chen, Haolin Song, Wei Xu, Jin Guo, Jianfei Wang, Juhong Zhou, Xiang Kang, Chaolei Jin, Yubo Cai, Zixuan Feng, Hainv Gao, Fengmin Lu, Lanjuan Li

**Affiliations:** ^1^ Department of Infectious Diseases, Shulan (Hangzhou) Hospital Affiliated to Zhejiang Shuren University Shulan International Medical College, Hangzhou, China; ^2^ College of Medicine, Zhejiang University, Hangzhou, China; ^3^ College of Basic Medical Sciences, Zhejiang Chinese Medical University, Hangzhou, China; ^4^ Shulan International Medical College, Zhejiang Shuren University, Hangzhou, China; ^5^ Research and Development Division, Oriomics Biotech Inc, Hangzhou, China; ^6^ Infection and Immunity Institute and Translational Medical Center of Huaihe Hospital, Henan University, Kaifeng, China; ^7^ State Key Laboratory for Diagnosis and Treatment of Infectious Diseases, First Affiliated Hospital, School of Medicine, Zhejiang University, Hangzhou, China; ^8^ National Clinical Research Center for Infectious Diseases, First Affiliated Hospital, School of Medicine, Zhejiang University, Hangzhou, China; ^9^ Collaborative Innovation Centre for Diagnosis and Treatment of Infectious Diseases, First Affiliated Hospital, College of Medicine, Zhejiang University, Hangzhou, China; ^10^ Department of Microbiology & Infectious Disease Center, School of Basic Medical Sciences, Peking University Health Science Center, Beijing, China; ^11^ Hepatology Institute, Peking University People’s Hospital, Beijing, China

**Keywords:** hepatocellular carcinoma, alpha-fetoprotein, DCP, GALAD, GAADPB

## Error in author list

In the published article, there was an error in the author list. The corrected author list appears below.

Ping Chen 1†, Haolin Song 2†, Wei Xu 3†, Jin Guo4, Jianfei Wang5, Juhong Zhou5, Xiang Kang5, Chaolei Jin6, Yubo Cai7,8,9, Zixuan Feng4, Hainv Gao1, Fengmin Lu10,11*, Lanjuan Li7,8,9*

The authors apologize for this error and state that this does not change the scientific conclusions of the article in any way. The original article has been updated.

## Incorrect affiliation

In the published article, there was an error in affiliation(s). Instead of

1State Key Laboratory for Diagnosis and Treatment of Infectious Diseases, First Affiliated Hospital, School of Medicine, Zhejiang University, Hangzhou, China,

2National Clinical Research Center for Infectious Diseases, First Affiliated Hospital, School of Medicine, Zhejiang University, Hangzhou, China,

3Collaborative Innovation Centre for Diagnosis and Treatment of Infectious Diseases, First Affiliated Hospital, College of Medicine, Zhejiang University, Hangzhou, China,

4Department of Microbiology & Infectious Disease Center, School of Basic Medical Sciences, Peking University Health Science Center, Beijing, China,

5Hepatology Institute, Peking University People’s Hospital, Beijing, China,

6Department of Infectious Diseases, Shulan (Hangzhou) Hospital Affiliated to Zhejiang Shuren University Shulan International Medical College, Hangzhou, China,

7College of Medicine, Zhejiang University, Hangzhou, China,

8College of Basic Medical Sciences, Zhejiang Chinese Medical University, Hangzhou, China,

9Shulan International Medical College, Zhejiang Shuren University, Hangzhou, China,

10Research and Development Division, Oriomics Biotech Inc, Hangzhou, China,

11Infection and Immunity Institute and Translational Medical Center of Huaihe Hospital, Henan University, Kaifeng, China,

it should be

1Department of Infectious Diseases, Shulan (Hangzhou) Hospital Affiliated to Zhejiang Shuren University Shulan International Medical College, Hangzhou, China,

2College of Medicine, Zhejiang University, Hangzhou, China,

3College of Basic Medical Sciences, Zhejiang Chinese Medical University, Hangzhou, China,

4Shulan International Medical College, Zhejiang Shuren University, Hangzhou, China,

5Research and Development Division, Oriomics Biotech Inc, Hangzhou, China,

6Infection and Immunity Institute and Translational Medical Center of Huaihe Hospital, Henan University, Kaifeng, China,

7State Key Laboratory for Diagnosis and Treatment of Infectious Diseases, First Affiliated Hospital, School of Medicine, Zhejiang University, Hangzhou, China,

8National Clinical Research Center for Infectious Diseases, First Affiliated Hospital, School of Medicine, Zhejiang University, Hangzhou, China,

9Collaborative Innovation Centre for Diagnosis and Treatment of Infectious Diseases, First Affiliated Hospital, College of Medicine, Zhejiang University, Hangzhou, China,

10Department of Microbiology & Infectious Disease Center, School of Basic Medical Sciences, Peking University Health Science Center, Beijing, China,

11Hepatology Institute, Peking University People’s Hospital, Beijing, China

The authors apologize for this error and state that this does not change the scientific conclusions of the article in any way. The original article has been updated.

