# Supplementary material for: Validation of the GALAD model and establishment of a new model for HCC detection in Chinese patients
Source: Front Oncol. 2022 Dec 23;12:1037742. doi: 10.3389/fonc.2022.1037742 (PMC9817025; doi:10.3389/fonc.2022.1037742)
Supplement: Supplementary file 1 [file DataSheet_1.docx]

**
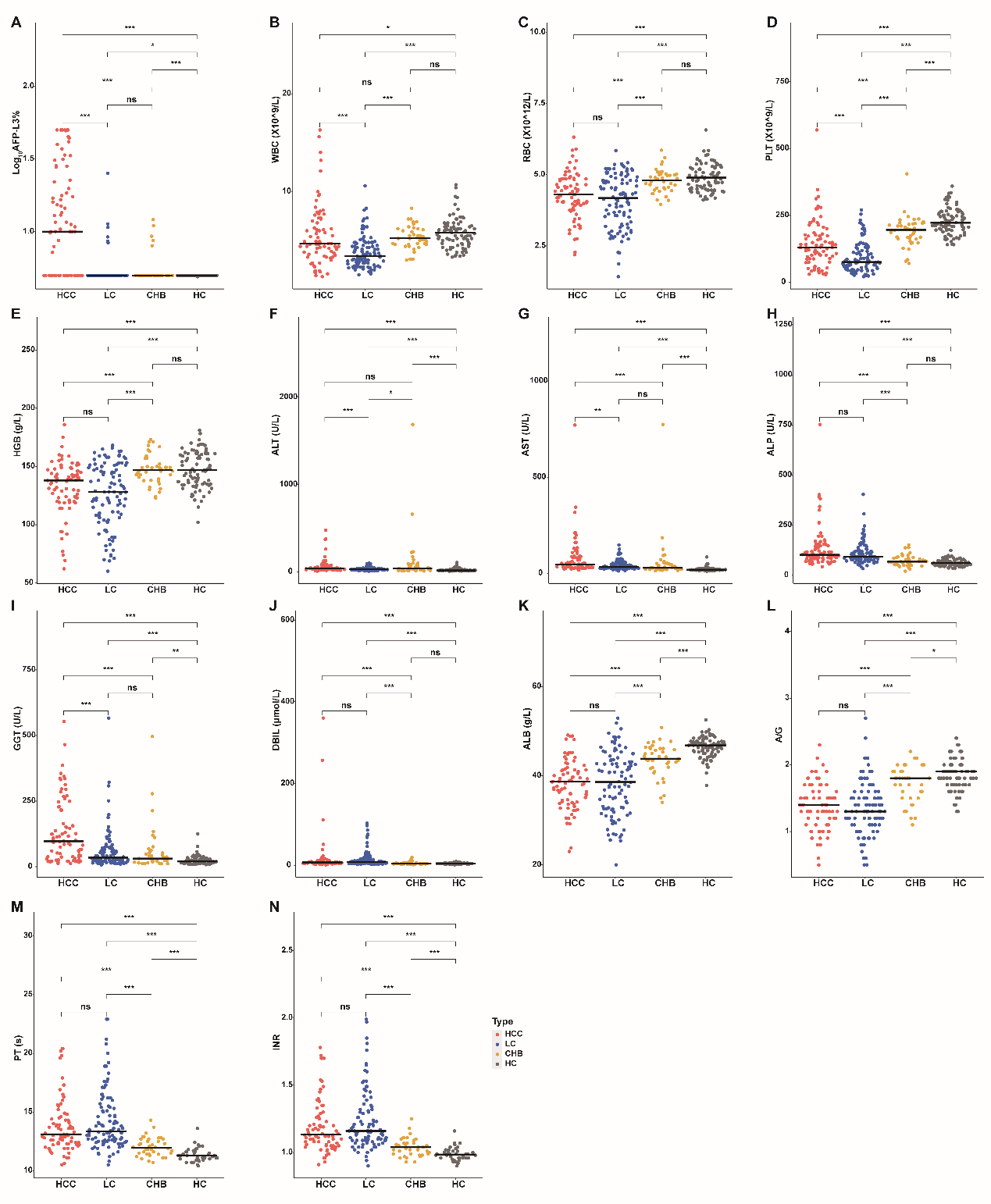
**

**Supplementary Fig. 1 Laboratory results in HCC group and non-HCC group.** Comparison of AFP-L3 (A), WBC (B), RBC (C), PLT (D), HGB (E), ALT (F), AST (G), ALP (H), GGT (I), DBIL (J), ALB (K), A/G (L), PT (M) and INR (D) among HCC, LC, CHB, and HC groups. The horizontal bar represents median values. Characteristics differences were tested using Wilcoxon test. ns *P*>0.05, **P* <0.05, ***P* <0.01, ****P* <0.001. Abbreviations: HCC, hepatocellular carcinoma; LC, liver cirrhosis; CHB, chronic hepatitis B; HC, healthy controls; AFP-L3, lens culinaris agglutinin-reactive alpha-fetoprotein; WBC, white blood cell; RBC, red blood cell; PLT, platelet; HGB, Hemoglobin; ALT, Alanine aminotransferase; AST, Aspartate aminotransferase; ALP, alkaline phosphatase; GGT, Gamma-glutamyl Transferase; DBIL, Direct Bilirubin; ALB, albumin; A/G, albumin/globulin (A/G) ratio; PT, Prothrombin time; INR, international normalized ratio.

| **Supplementary Table 1 Characteristics of the study participants** | | | | | |  |
| --- | --- | --- | --- | --- | --- | --- |
| **Variables** | **HCC(n=80)** | **CLD** | | | **HC(n=87)** | ***P* value (HCC vs CLD)** |
|  |  | **Total(n=140)** | **Cirrhosis(n=100)** | **CHB（n=40）** |  |  |
| **HCC biomarkers** |  |  |  |  |  |  |
| AFP-L3, (%) | 10(5~22.2) | 5(5~5) | 5(5~5) | 5(5~5) | 5(5~5) | <0.001^***^ |
| **Blood routine examination** |  |  |  |  |  |  |
| WBC, (10E9/L) | 4.7(3.6~6.9) | 4.1(2.9~5.2) | 3.4(2.6~4.6) | 5.3(4.6~6.2) | 5.8(4.7~6.5) | 0.004^**^ |
| RBC, (10E12/L) | 4.3(3.9~4.8) | 4.5(3.7~5) | 4.2(3.3~4.8) | 4.8(4.5~5) | 4.9(4.5~5.3) | 0.497 |
| PLT, (10E9/L) | 130(87.8~177.8) | 102.5(63.8~177) | 75(54.8~119.8) | 195.5(161.8~219.3) | 222(194.5~259.5) | 0.039^*^ |
| HGB, (g/L) | 138(122.5~147.3) | 138(117~150.3) | 128(106~150) | 147(138.8~153.3) | 147(135~161) | 0.661 |
| **Liver function tests** |  |  |  |  |  |  |
| ALT, (U/L) | 41(24.8~66) | 31(21.5~45.5) | 28.5(21~38.3) | 39(23~82.5) | 17(13~28.5) | 0.01^*^ |
| AST, (U/L) | 47(31~84.8) | 32(26~52) | 33(27~52.3) | 29(21~51.5) | 19(17~23.5) | <0.001^***^ |
| ALP, (U/L) | 102(84.5~148) | 86(68~114) | 93(77~122.8) | 68(51.5~87) | 62(51~76) | <0.001^***^ |
| GGT, (U/L) | 97(37~215) | 34(20.5~63.5) | 34.5(21~66.75) | 31(17.5~55.5) | 20(14.5~31) | <0.001^***^ |
| DBIL, (μmol/L) | 7(4.75~10.3) | 7(4~12) | 8(5~16.25) | 4(3~6) | 4(3~5) | 0.883 |
| ALB, (g/L) | 38.7(34.4~41.3) | 41.6(35.1~45) | 38.6(32.8~43.5) | 43.8(41.9~46.1) | 46.8(45.3~47.7) | 0.017^*^ |
| A/G | 1.4(1.1~1.7) | 1.4(1.2~1.8) | 1.3(1.1~1.6) | 1.8(1.6~1.9) | 1.9(1.7~2) | 0.211 |
| **Blood coagulation function** |  |  |  |  |  |  |
| PT, (s) | 13.1(12.3~14.4) | 12.8(12.1~14.3) | 13.4(12.5~15.4) | 12(11.4~12.7) | 11.3(11~11.6) | 0.363 |
| INR | 1.1(1.1~1.3) | 1.1(1.1~1.2) | 1.2(1.1~1.3) | 1(1~1.1) | 1(1~1) | 0.315 |

Note: All continuous variables were presented as median (interquartile range). Abbreviations: HCC, hepatocellular carcinoma; CLD, chronic liver disease; LC, liver cirrhosis; CHB, chronic hepatitis B; HC, healthy controls; AFP-L3, lens culinaris agglutinin-reactive alpha-fetoprotein; WBC, white blood cell; RBC, red blood cell; PLT, platelet; HGB, Hemoglobin; ALT, Alanine aminotransferase; AST, Aspartate aminotransferase; ALP, alkaline phosphatase; GGT, Gamma-glutamyl Transferase; DBIL, Direct Bilirubin; ALB, albumin; A/G, albumin/globulin (A/G) ratio; PT, Prothrombin time; INR, international normalized ratio.

**Supplementary Table 2 Equations for five HCC risk assessment models**

| **Model Name** | **Equation** | **Cutoff value** |
| --- | --- | --- |
| **GALAD** | Z=-10.08+0.09*age+1.67*gender+2.34*log_10_AFP+0.04*AFP-L3+1.33*log_10_DCP | -0.63 |
| **GAAP** | Score=-11.203+0.699*gender+0.094*age+1.076*log_10_AFP+2.376*log_10_DCP | -0.65 |
| **Doylestown** | P=1/(1+exp(-[-10.307+0.097*age+1.645*Gender+2.315*log_10_AFP+0.011*ALP-0.008*ALT])) | 0.5 |
| **BALAD-2** | Linear predictor=0.02*(AFP-2.57)+0.012*[(AFP-L3)-14.19]+0.19*[lnDCP-1.93]+0.17*[(bilirubin)^1/2^-4.5]-0.09*(albumin-35.11) | 0.66 |
| **aMAP** | Score=((0.06*age+0.89*gender+0.48*(0.66*log10bilirubin-0.085*albumin)-0.01*PLT)+7.4)/14.77*100 | 60 |

Note: BALAD-2: AFP and DCP modeled as / 1000 units. Units: AFP(ng/ml), DCP(ng/ml), AFP-L3(%), bilirubin(μmol/L), albumin(g/L), ALT(U/L), ALP(U/L), PLT(10^3^/mm^3^); Gender: 1 for male and 0 for female.

**Supplementary Table 3 Comparison between GAADPB, GALAD and the individual biomarkers within cancer subgroups in training set (Specificity=90%)**

| **Cancer subgroups** | **AUC (95%CI)** | | | | **Sensitivity %** | | | | ***P* value  (GAADPB VS others)** | | |
| --- | --- | --- | --- | --- | --- | --- | --- | --- | --- | --- | --- |
|  | **GAADPB** | **GALAD** | **AFP** | **DCP** | **GAADPB** | **GALAD** | **AFP** | **DCP** | **GALAD** | **AFP** | **DCP** |
| **Tumor stage (BCLC)** | | | | | | | | | | | |
| 0/A | 0.916 (0.866~0.966) | 0.904 (0.856~0.952) | 0.856 (0.78~0.932) | 0.729 (0.634~0.823) | 78.8 | 66.7 | 60.6 | 39.4 | 0.349 | 0.009^**^ | <0.001^***^ |
| B/C/D | 0.991 (0.983~0.999) | 0.982 (0.97~0.995) | 0.94 (0.902~0.978) | 0.963 (0.932~0.994) | 97.9 | 95.7 | 76.6 | 87.2 | 0.015^*^ | 0.003^**^ | 0.062 |
| **Tumor size** | | | | | | | | | | | |
| ＜3cm | 0.926 (0.875~0.976) | 0.91 (0.863~0.957) | 0.878 (0.801~0.955) | 0.689 (0.574~0.805) | 79.2 | 66.7 | 58.3 | 37.5 | 0.352 | 0.022^*^ | <0.001^***^ |
| ≥3 and≤5cm | 0.94 (0.877~1) | 0.936 (0.873~0.999) | 0.871 (0.771~0.971) | 0.903 (0.83~0.975) | 88.9 | 83.3 | 66.7 | 66.7 | 0.54 | 0.029^*^ | 0.036^*^ |
| ＞5cm | 0.992 (0.982~1) | 0.986 (0.97~1) | 0.935 (0.876~0.995) | 0.956 (0.906~1) | 96.3 | 96.3 | 81.5 | 85.2 | 0.111 | 0.039^*^ | 0.151 |
| **Number of tumors** | | | | | | | | | | | |
| single | 0.925 (0.877~0.972) | 0.916 (0.871~0.96) | 0.862 (0.789~0.934) | 0.782 (0.695~0.868) | 80 | 71.4 | 57.1 | 48.6 | 0.44 | 0.003^**^ | <0.001^***^ |
| multiple | 0.988 (0.978~0.998) | 0.977 (0.96~0.994) | 0.941 (0.901~0.981) | 0.93 (0.879~0.981) | 97.7 | 93 | 81.4 | 81.4 | 0.027^*^ | 0.011^*^ | 0.017^*^ |
| **PPVT** | | | | | | | | | | | |
| absent | 0.943 (0.91~0.976) | 0.932 (0.899~0.965) | 0.876 (0.82~0.932) | 0.822 (0.755~0.889) | 85.2 | 77.8 | 64.8 | 55.6 | 0.181 | 0.001^**^ | <0.001^***^ |
| present | 0.995 (0.99~1) | 0.987 (0.975~1) | 0.966 (0.942~0.991) | 0.958 (0.906~1) | 100 | 96.2 | 80.8 | 92.3 | 0.08 | 0.009^**^ | 0.157 |
| **Metastasis** | | | | | | | | | | | |
| absent | 0.955 (0.929~0.981) | 0.944 (0.918~0.971) | 0.893 (0.847~0.939) | 0.857 (0.803~0.912) | 88.6 | 81.4 | 65.7 | 64.3 | 0.107 | <0.001^***^ | <0.001^***^ |
| present | 0.996 (0.987~1) | 0.99 (0.977~1) | 0.99 (0.976~1) | 0.928 (0.792~1) | 100 | 100 | 100 | 90 | 0.223 | 0.359 | 0.304 |
| **AFP** | | | | | | | | | | | |
| <20 ng/ml | 0.917 (0.866~0.968) | 0.894 (0.846~0.943) |  |  | 75 | 62.5 |  |  | 0.088 |  |  |
| 20-400 ng/ml | 0.976 (0.959~0.994) | 0.972 (0.946~0.998) |  |  | 100 | 95.2 |  |  | 0.611 |  |  |
| >400 ng/ml | 0.999 (0.997~1) | 0.999 (0.997~1) |  |  | 100 | 100 |  |  | 0.825 |  |  |
| **DCP** | | | | | | | | | | | |
| <40 ng/ml | 0.894 (0.833~0.954) | 0.883 (0.825~0.941) |  |  | 69.2 | 61.5 |  |  | 0.496 |  |  |
| ≥40 ng/ml | 0.992 (0.985~0.999) | 0.983 (0.971~0.994) |  |  | 100 | 94.4 |  |  | 0.011* |  |  |

Note: “/”, not calculated.

Abbreviations: AUC, area under receiver operating characteristic curve; PPVT, portal vein tumor thrombus; AFP, alpha-fetoprotein; DCP, des-gamma-carboxy prothrombin.

| **Supplementary Table 4 Comparison between GAADPB and the individual biomarkers within cancer subgroups in test set (Specificity=90%)** | | | | | | | | | | | |
| --- | --- | --- | --- | --- | --- | --- | --- | --- | --- | --- | --- |
| **Cancer subgroups** | **AUC(95%CI)** | | | | **Sensitivity %** | | | | ***P* value**  **(GAADPB VS others)** | | |
|  | **GAADPB** | **GALAD** | **AFP** | **DCP** | **GAADPB** | **GALAD** | **AFP** | **DCP** | **GALAD** | **AFP** | **DCP** |
| **Tumor stage(BCLC)** |  |  |  |  |  |  |  |  |  |  |  |
| 0/A | 0.838 | 0.841 | 0.758 | 0.758 | 56.3 | 53.1 | 40.6 | 50 | 0.9 | 0.034^*^ | 0.028^*^ |
|  | (0.765~0.91) | (0.768~0.914) | (0.665~0.851) | (0.66~0.856) |  |  |  |  |  |  |  |
| B/C/D | 0.94 | 0.927 | 0.88 | 0.883 | 90.2 | 82.9 | 75.6 | 82.9 | 0.355 | 0.051 | 0.024^*^ |
|  | (0.894~0.987) | (0.872~0.982) | (0.807~0.954) | (0.808~0.957) |  |  |  |  |  |  |  |
| **Tumor size** |  |  |  |  |  |  |  |  |  |  |  |
| ＜3cm | 0.819 | 0.792 | 0.712 | 0.691 | 52.2 | 39.1 | 26.1 | 34.8 | 0.461 | 0.032^*^ | 0.01^*^ |
|  | (0.737~0.902) | (0.701~0.883) | (0.6~0.823) | (0.572~0.81) |  |  |  |  |  |  |  |
| ≥3 and≤5cm | 0.933 | 0.96 | 0.929 | 0.917 | 85.7 | 85.7 | 85.7 | 85.7 | 0.157 | 0.764 | 0.794 |
|  | (0.837~1) | (0.895~1) | (0.831~1) | (0.82~1) |  |  |  |  |  |  |  |
| ＞5cm | 0.948 | 0.933 | 0.854 | 0.916 | 86.4 | 86.4 | 72.7 | 86.4 | 0.423 | 0.043^*^ | 0.174 |
|  | (0.898~0.999) | (0.86~1) | (0.737~0.971) | (0.832~1) |  |  |  |  |  |  |  |
| **Number of tumors** |  |  |  |  |  |  |  |  |  |  |  |
| single | 0.843 | 0.84 | 0.76 | 0.781 | 57.1 | 50 | 39.3 | 50 | 0.904 | 0.039^*^ | 0.087 |
|  | (0.767~0.92) | (0.762~0.917) | (0.662~0.859) | (0.684~0.879) |  |  |  |  |  |  |  |
| multiple | 0.928 | 0.92 | 0.868 | 0.857 | 86.7 | 82.2 | 73.3 | 80 | 0.636 | 0.04^*^ | 0.007^**^ |
|  | (0.88~0.975) | (0.867~0.974) | (0.796~0.94) | (0.779~0.935) |  |  |  |  |  |  |  |
| **PPVT** |  |  |  |  |  |  |  |  |  |  |  |
| absent | 0.855 | 0.842 | 0.764 | 0.754 | 63 | 56.5 | 45.7 | 54.3 | 0.587 | 0.004^**^ | 0.003^**^ |
|  | (0.794~0.915) | (0.775~0.909) | (0.681~0.846) | (0.666~0.841) |  |  |  |  |  |  |  |
| present | 0.964 | 0.97 | 0.934 | 0.955 | 96.3 | 92.6 | 85.2 | 92.6 | 0.584 | 0.434 | 0.236 |
|  | (0.913~1) | (0.935~1) | (0.864~1) | (0.904~1) |  |  |  |  |  |  |  |
| **Metastasis** |  |  |  |  |  |  |  |  |  |  |  |
| absent | 0.884 | 0.872 | 0.796 | 0.802 | 71.2 | 64.4 | 54.2 | 62.7 | 0.533 | 0.003^**^ | 0.002^**^ |
|  | (0.834~0.934) | (0.817~0.928) | (0.724~0.869) | (0.729~0.876) |  |  |  |  |  |  |  |
| present | 0.942 | 0.96 | 0.954 | 0.936 | 92.9 | 92.9 | 85.7 | 92.9 | 0.358 | 0.704 | 0.478 |
|  | (0.846~1) | (0.901~1) | (0.91~0.999) | (0.846~1) |  |  |  |  |  |  |  |
| **AFP** |  |  |  |  |  |  |  |  |  |  |  |
| <20 ng/ml | 0.78 | 0.764 |  |  | 42.4 | 33.3 |  |  | 0.645 | 0.005^**^ | 0.013^*^ |
|  | (0.699~0.861) | (0.679~0.849) |  |  |  |  |  |  |  |  |  |
| 20-400 ng/ml | 0.981 | 0.976 |  |  | 100 | 94.7 |  |  | 0.725 | 0.409 | 0.067 |
|  | (0.961~1) | (0.941~1) |  |  |  |  |  |  |  |  |  |
| >400 ng/ml | 0.992 | 0.992 |  |  | 100 | 100 |  |  | 1 | 0.334 | 0.211 |
|  | (0.977~1) | (0.977~1) |  |  |  |  |  |  |  |  |  |
| **DCP** |  |  |  |  |  |  |  |  |  |  |  |
| <40 ng/ml | 0.74 | 0.723 |  |  | 36.4 | 27.3 |  |  | 0.722 | 0.335 | 0.015^*^ |
|  | (0.639~0.842) | (0.614~0.833) |  |  |  |  |  |  |  |  |  |
| ≥40 ng/ml | 0.961 | 0.956 |  |  | 90.6 | 86.8 |  |  | 0.543 | 0.004^**^ | 0.591 |
|  | (0.933~0.988) | (0.927~0.985) |  |  |  |  |  |  |  |  |  |

Note: “/”, not calculated.

Abbreviations: AUC, area under receiver operating characteristic curve; PPVT, portal vein tumor thrombus; AFP, alpha-fetoprotein; DCP, des-gamma-carboxy prothrombin.
